# Supplementary material for: Bayesian approach to assessing population differences in genetic risk of disease with application to prostate cancer
Source: PLoS Genet. 2024 Apr 17;20(4):e1011212. doi: 10.1371/journal.pgen.1011212 (PMC11023298; doi:10.1371/journal.pgen.1011212)
Supplement: S1 Appendix — (DOCX) [file pgen.1011212.s001.docx]

**S1 Appendix**

**Analytical form of posterior mean and variance of SNP effect sizes under non-infinitesimal model.**

The prior distribution is defined as:

$$\beta_{j}\sim\left\{ \begin{aligned} \begin{matrix} N\left( 0,v_{j}^{2} \right), & \text{with probability} p_{\text{causal}} \end{matrix} \\ \begin{matrix} 0 & \text{ , }\text{ }\text{ }\text{with probability} 1-p_{\text{causal}} \end{matrix} \end{aligned} \right.$$

where $v_{j}^{2}=\frac{h_{g}^{2}}{Mp_{\text{causal}}\left[ 2f_{j}\left( 1-f_{j} \right) \right]}$

The marginal GWAS effect estimates have the approximate distribution:

$$\hat{\beta}_{\text{GWAS}, j}|\beta_{j} \sim N\left( \beta_{j},\tau_{j}^{2} \right)$$

where $\tau_{j}^{2}=\frac{1}{2{Nf}_{j}(1-f_{j})}$

We are interested in the posterior mean, which can be expressed as

$$\mathbb{E[}\beta_{j}|\hat{\beta}_{\text{GWAS}, j}]=\int_{-\infty}^{\infty} \frac{\beta_{j}f(\hat{\beta}_{\text{GWAS}, j}|\beta_{j})f(\beta_{j})}{\int_{-\infty}^{\infty} f(\hat{\beta}_{\text{GWAS}, j}|\beta_{j})f(\beta_{j})d\beta_{j}}{d\beta}_{j}$$

And the posterior variance

$$\text{var}[\beta_{j}|\hat{\beta}_{\text{GWAS}, j}]=\int_{-\infty}^{\infty} \frac{\beta_{j}^{2}f(\hat{\beta}_{\text{GWAS}, j}|\beta_{j})f(\beta_{j})}{\int_{-\infty}^{\infty} f(\hat{\beta}_{\text{GWAS}, j}|\beta_{j})f(\beta_{j})d\beta_{j}}{d\beta}_{j}-{(\mathbb{E[}\beta_{j}|\hat{\beta}_{\text{GWAS}, j}])}^{2}$$

The prior distribution has density function

$$f\left( \beta_{j} \right)=p_{\text{causal}}\varphi_{N\left( 0,v_{j}^{2} \right)}\left( \beta_{j} \right)+(1-p_{\text{causal}})\delta_{\beta_{j}}$$

where $\delta$ is the Dirac delta function. The marginal effect estimates have density function:

$$f\left( \hat{\beta}_{\text{GWAS}, j}|\beta_{j} \right)=\varphi_{N\left( \beta_{j},\tau_{j}^{2} \right)}\left( \hat{\beta}_{\text{GWAS}, j} \right)$$

Note that:

$$\varphi_{N\left( 0,v_{j}^{2} \right)}\left( \beta_{j} \right)\varphi_{N\left( \beta_{j},\tau_{j}^{2} \right)}\left( \hat{\beta}_{\text{GWAS}, j} \right)=\left( \frac{1}{2\pi\sqrt{v_{j}^{2} \tau_{j}^{2}}}exp\left[ -\frac{\hat{\beta}_{\text{GWAS},j}^{2}}{2\tau_{j}^{2}} \right] \right)\exp\left[ -\frac{1}{2}\left( \frac{1}{\tau_{j}^{2}}+\frac{1}{v_{j}^{2}} \right)\beta_{j}^{2}+\frac{\hat{\beta}_{\text{GWAS}, j}}{\tau_{j}^{2}}\beta_{j} \right]$$

Hence, let

$$A_{j}=\frac{1}{2}\left( \frac{1}{\tau_{j}^{2}}+\frac{1}{v_{j}^{2}} \right)$$

$$B_{j}=\frac{\hat{\beta}_{\text{GWAS}, j}}{\tau_{j}^{2}}$$

$$C_{j}=\frac{1}{2\pi\sqrt{v_{j}^{2} \tau_{j}^{2}}}exp\left[ -\frac{\hat{\beta}_{\text{GWAS},j}^{2}}{2\tau_{j}^{2}} \right]$$

We note the following results for exponential integrals [1]:

$$\int_{-\infty}^{\infty} exp[-A\beta^{2}+B\beta]d\beta=\sqrt{\frac{\pi}{A}}exp\left[ \frac{B^{2}}{4A} \right]$$

$$\int_{-\infty}^{\infty} \beta exp[-A\beta^{2}+B\beta]d\beta=\frac{B\sqrt{\pi}}{{2A}^{3/2}}exp\left[ \frac{B^{2}}{4A} \right]$$

$$\int_{-\infty}^{\infty} \beta^{2}exp[-A\beta^{2}+B\beta]d\beta=\frac{(2A+B^{2})\sqrt{\pi}}{4A^{5/2}}exp\left[ \frac{B^{2}}{4A} \right]$$

First, we have the integral:

$$I_{1}=\int_{-\infty}^{\infty} f(\hat{\beta}_{\text{GWAS}, j}|\beta_{j})f(\beta_{j})d\beta_{j}$$

$$=\int_{-\infty}^{\infty} \varphi_{N\left( \beta_{j},\tau_{j}^{2} \right)}\left( \hat{\beta}_{\text{GWAS}, j} \right)(p_{\text{causal}}\varphi_{N\left( 0,v_{j}^{2} \right)}\left( \beta_{j} \right)+(1-p_{\text{causal}})\delta_{\beta_{j}})d\beta_{j}$$

$$=p_{\text{causal}}\int_{-\infty}^{\infty} \varphi_{N\left( \beta_{j},\tau_{j}^{2} \right)}\left( \hat{\beta}_{\text{GWAS}, j} \right)\varphi_{N\left( 0,v_{j}^{2} \right)}\left( \beta_{j} \right)d\beta_{j}+(1-p_{\text{causal}})\varphi_{N\left( 0,\tau_{j}^{2} \right)}\left( \hat{\beta}_{\text{GWAS}, j} \right)$$

$$=p_{\text{causal}}C_{j}\int_{-\infty}^{\infty} exp[-A_{i}\beta_{j}^{2}+B_{i}\beta_{j}]d\beta_{j}+(1-p_{\text{causal}})C_{j}\sqrt{2\pi v_{j}^{2}}$$

$$=C_{j}\left\{ p_{\text{causal}}\sqrt{\frac{\pi}{A_{j}}}exp\left[ \frac{B_{j}^{2}}{4A_{j}} \right]+(1-p_{\text{causal}})\sqrt{2\pi\tau_{j}^{2}} \right\}$$

Second,

$$I_{2}=\int_{-\infty}^{\infty} \beta_{j}f(\hat{\beta}_{\text{GWAS}, j}|\beta_{j})f(\beta_{j})d\beta_{j}$$

$$=\int_{-\infty}^{\infty} \beta_{j}\varphi_{N\left( \beta_{j},\tau_{j}^{2} \right)}\left( \hat{\beta}_{\text{GWAS}, j} \right)(p_{\text{causal}}\varphi_{N\left( 0,v_{j}^{2} \right)}\left( \beta_{j} \right)+(1-p_{\text{causal}})\delta_{\beta_{j}})d\beta_{j}$$

$$=p_{\text{causal}}\int_{-\infty}^{\infty} \beta_{j}\varphi_{N\left( \beta_{j},\tau_{j}^{2} \right)}\left( \hat{\beta}_{\text{GWAS}, j} \right)\varphi_{N\left( 0,v_{j}^{2} \right)}\left( \beta_{j} \right)d\beta_{j}$$

$$=p_{\text{causal}}C_{j}\int_{-\infty}^{\infty} \beta_{j} exp[-A_{i}\beta_{j}^{2}+B_{i}\beta_{j}]d\beta_{j}$$

$$=p_{\text{causal}}C_{j}\frac{B_{j}\sqrt{\pi}}{2A_{j}^{3/2}}exp\left[ \frac{B_{j}^{2}}{4A_{j}} \right]$$

And third,

$$I_{3}=\int_{-\infty}^{\infty} \beta_{j}^{2}f(\hat{\beta}_{\text{GWAS}, j}|\beta_{j})f(\beta_{j})d\beta_{j}$$

$$=\int_{-\infty}^{\infty} {\beta_{j}^{2}\varphi}_{N\left( \beta_{j},\tau_{j}^{2} \right)}\left( \hat{\beta}_{\text{GWAS}, j} \right)(p_{\text{causal}}\varphi_{N\left( 0,v_{j}^{2} \right)}\left( \beta_{j} \right)+(1-p_{\text{causal}})\delta_{\beta_{j}})d\beta_{j}$$

$$=p_{\text{causal}}\int_{-\infty}^{\infty} {\beta_{j}^{2}\varphi}_{N\left( \beta_{j},\tau_{j}^{2} \right)}\left( \hat{\beta}_{\text{GWAS}, j} \right)\varphi_{N\left( 0,v_{j}^{2} \right)}\left( \beta_{j} \right)d\beta_{j}$$

$$=p_{\text{causal}}C_{j}\int_{-\infty}^{\infty} \beta_{j}^{2} exp[-A_{i}\beta_{j}^{2}+B_{i}\beta_{j}]d\beta_{j}$$

$$=p_{\text{causal}}C_{j}\frac{(2A_{i}+B_{j}^{2})\sqrt{\pi}}{4A_{j}^{5/2}}exp\left[ \frac{B_{j}^{2}}{4A_{j}} \right]$$

Hence, the posterior mean can be expressed as:

$$\mathbb{E[}\beta_{j}|\hat{\beta}_{\text{GWAS}, j}]=\frac{I_{2}}{I_{1}}$$

$$=\frac{p_{\text{causal}}C_{j}\frac{B_{j}\sqrt{\pi}}{A_{j}^{3/2}}exp\left[ \frac{B_{j}^{2}}{4A_{j}} \right]}{C_{j}\left\{ p_{\text{causal}}\sqrt{\frac{\pi}{A_{j}}}exp\left[ \frac{B_{j}^{2}}{4A_{j}} \right]+(1-p_{\text{causal}})\sqrt{2\pi\tau_{j}^{2}} \right\}}$$

$$=\frac{p_{\text{causal}}\frac{B_{j}\sqrt{\pi}}{2A_{j}^{3/2}}}{p_{\text{causal}}\sqrt{\frac{\pi}{A_{j}}}+(1-p_{\text{causal}})\sqrt{2\pi\tau_{j}^{2}}exp\left[ -\frac{B_{j}^{2}}{4A_{j}} \right]}$$

While the posterior variance can be expressed as:

$$\text{var}[\beta_{j}|\hat{\beta}_{\text{GWAS}, j}]=\frac{I_{3}}{I_{1}}-{\mathbb{(E[}\beta_{j}|\hat{\beta}_{\text{GWAS}, j}])}^{2}$$

$$=\frac{p_{\text{causal}}C_{j}\frac{(2A_{j}+B_{j}^{2})\sqrt{\pi}}{4A_{j}^{5/2}}exp\left[ \frac{B_{j}^{2}}{4A_{j}} \right]}{C_{j}\left\{ p_{\text{causal}}\sqrt{\frac{\pi}{A_{j}}}exp\left[ \frac{B_{j}^{2}}{4A_{j}} \right]+(1-p_{\text{causal}})\sqrt{2\pi\tau_{j}^{2}} \right\}}-{\mathbb{(E[}\beta_{j}|\hat{\beta}_{\text{GWAS}, j}])}^{2}$$

$$=\left( \frac{p_{\text{causal}}\frac{(2A_{j}+B_{j}^{2})\sqrt{\pi}}{4A_{j}^{5/2}}}{p_{\text{causal}}\sqrt{\frac{\pi}{A_{j}}}+(1-p_{\text{causal}})\sqrt{2\pi\tau_{j}^{2}}exp\left[ -\frac{B_{j}^{2}}{4A_{j}} \right]} \right)-\left( \frac{p_{\text{causal}}\frac{B_{j}\sqrt{\pi}}{2A_{j}^{3/2}}}{p_{\text{causal}}\sqrt{\frac{\pi}{A_{j}}}+(1-p_{\text{causal}})\sqrt{2\pi\tau_{j}^{2}}exp\left[ -\frac{B_{j}^{2}}{4A_{j}} \right]} \right)^{2}$$

The posterior mean and variance of $\beta_{j}|\hat{\beta}_{\text{GWAS}, j}$ can thus be expressed in terms of $p_{\text{causal}}$, $\tau_{j}^{2}$, $A_{j}$, $B_{j}$, which means these expressions can be further related back in terms of $\hat{\beta}_{\text{GWAS}, j}$, along with the original parameters $h_{g}^{2}$, $p_{\text{causal}}$, $f_{j}$, $M$ and $N$.

**References**

1. Moll VH. Special Integrals of Gradshteyn and Ryzhik. New York: Chapman and Hall/CRC; 2015.
