# Supplementary material for: Bayesian approach to assessing population differences in genetic risk of disease with application to prostate cancer
Source: PLoS Genet. 2024 Apr 17;20(4):e1011212. doi: 10.1371/journal.pgen.1011212 (PMC11023298; doi:10.1371/journal.pgen.1011212)
Supplement: S2 Appendix — (DOCX) [file pgen.1011212.s002.docx]

## S2 Appendix

## Closed-form expression for posterior variance under infinitesimal model.

To understand the uncertainty in the estimate of $d$, we derive an analytical estimate of the posterior distribution of $d$ under the assumptions that all SNPs are causal, and are i.i.d. and drawn from:

$$\beta_{j}\sim N\left( 0,\frac{h_{g}^{2}}{M\left[ 2f_{j}\left( 1-f_{j} \right) \right]} \right)$$

for $i$ in 1, …, $M$. As shown by Daetwyler et al. [1], the least squares estimate of the GWAS marginal effect $\hat{\beta}_{\mathrm{GWAS},j}$ is approximately distributed as

$$\hat{\beta}_{\text{GWAS}, j}|\beta_{j} \sim N\left( \beta_{j}, \frac{1}{2{Nf}_{j}(1-f_{j})}\left( 1-\frac{h_{g}^{2}}{M} \right) \right)$$

The per-SNP heritability is assumed small, hence the $\left( 1-\frac{h_{g}^{2}}{M} \right)$ term can be replaced with unity. The posterior distribution of $\beta_{j}|\hat{\beta}_{\text{GWAS}, j}$ can be approximated as

$$\beta_{j}|\hat{\beta}_{\text{GWAS}, j} \sim N\left( \left( 1+\frac{M}{h_{g}^{2}N} \right)^{-1}\hat{\beta}_{\text{GWAS}, j}, \frac{1}{2{Nf}_{j}(1-f_{j})}\left( 1+\frac{M}{Nh_{g}^{2}} \right)^{-1} \right)$$

The posterior distribution of $d|{\hat{\boldsymbol{\beta}}}_{\text{GWAS}}$ becomes

$$d|{\hat{\boldsymbol{\beta}}}_{\text{GWAS}}\boldsymbol{\sim N}\left( \left( 1+\frac{M}{h_{g}^{2}N} \right)^{-1} \left( \sum_{1}^{M} 2\left( f_{j}-g_{j} \right)\hat{\beta}_{\text{GWAS}, j} \right), \frac{4MF_{ST}}{N}\left( 1+\frac{M}{Nh_{g}^{2}} \right)^{-1} \right)$$

where we use the result that (see S4 Appendix), under the Balding-Nichols model [2], the Wright $F_{ST}$statistic can be approximated as

$$F_{ST}\boldsymbol{\approx}\frac{1}{M}\sum_{1}^{M} \frac{\left( f_{j}-g_{j} \right)^{2}}{2f_{j}\left( 1-f_{j} \right)}$$

Hence, the posterior variance of $d$ can be approximated as

$$\text{var}[d|{\hat{\boldsymbol{\beta}}}_{\text{GWAS}}\boldsymbol{,}h_{g}^{2}] = \frac{4MF_{ST}}{N}\left( 1+\frac{M}{Nh_{g}^{2}} \right)^{-1}$$

## References

1. Daetwyler HD, Villanueva B, Woolliams JA. Accuracy of predicting the genetic risk of disease using a genome-wide approach. PLoS One. 2008;3(10):e3395. Epub 2008/10/15. doi: 10.1371/journal.pone.0003395. PubMed PMID: 18852893; PubMed Central PMCID: PMCPMC2561058.

2. Balding DJ, Nichols RA. A method for quantifying differentiation between populations at multi-allelic loci and its implications for investigating identity and paternity. Genetica. 1995;96(1-2):3-12. Epub 1995/01/01. doi: 10.1007/BF01441146. PubMed PMID: 7607457.
