# Supplementary material for: Bayesian approach to assessing population differences in genetic risk of disease with application to prostate cancer
Source: PLoS Genet. 2024 Apr 17;20(4):e1011212. doi: 10.1371/journal.pgen.1011212 (PMC11023298; doi:10.1371/journal.pgen.1011212)
Supplement: S3 Appendix — (DOCX) [file pgen.1011212.s003.docx]

## S3 Appendix

## Wald test on the posterior mean estimator $\hat{\boldsymbol{d}}\mathbb{=E[}\boldsymbol{d|}{\hat{\boldsymbol{\beta}}}_{\mathbf{GWAS}}\boldsymbol{;}\boldsymbol{h}_{\boldsymbol{g}}^{\boldsymbol{2}}\boldsymbol{,}\boldsymbol{p}_{\text{causal}}\boldsymbol{]}$

The posterior mean $\hat{d}$ is given by

$$\hat{d}=\sum_{1}^{M} 2(f_{j}-g_{j})\mathbb{E[}\beta_{j}|\hat{\beta}_{\text{GWAS}, j};h_{g}^{2},p_{\text{causal}}]$$

We consider the Wald statistic:

$$W=\left( \frac{\hat{d}}{s.e.(\hat{d})} \right)^{2}$$

Under the null hypothesis $d=0$, the Wald statistic follows an asymptotic $\chi^{2}$-distribution with one degree of freedom. To evaluate this statistic, we derive the standard error of $\hat{d}$.

Using the results and notation from S1 Appendix, recall the definitions:

$$v_{j}^{2}=\frac{h_{g}^{2}}{Mp_{\text{causal}}\left[ 2f_{j}\left( 1-f_{j} \right) \right]}$$

$$\tau_{j}^{2}=\frac{1}{2{Nf}_{j}(1-f_{j})}$$

$$A_{j}=\frac{1}{2}\left( \frac{1}{\tau_{j}^{2}}+\frac{1}{v_{j}^{2}} \right)$$

Defining new constants $A_{2,j}$, $B_{2.j}$, $C_{2,j}$ (that are not dependent on $\hat{\beta}_{\text{GWAS}, j}$):

$$A_{2,j}=p_{\text{causal}}\frac{1}{2A_{j}^{3/2}\tau_{j}^{2}\sqrt{2v_{j}^{2}}}$$

$$B_{2,j}=p_{\text{causal}}\frac{1}{A_{j}^{1/2}\sqrt{2\pi v_{j}^{2}}}$$

$$C_{2,j}=\frac{1}{4A_{j}\tau_{j}^{4}}$$

We can write the posterior mean of SNP effects sizes in the following form:

$$\mathbb{E[}\beta_{j}|\hat{\beta}_{\text{GWAS}, j}]=\frac{A_{2,j}\hat{\beta}_{\text{GWAS}, j}}{B_{2,j}+(1-p_{\text{causal}})exp\left[ -{C_{2,j}\hat{\beta}}_{\text{GWAS},j}^{2} \right]}$$

Next, we apply the delta method to the function $h_{j}$, where we have:

$$h_{j}\left( \hat{\beta}_{\text{GWAS}, j} \right)=\frac{A_{2,j}\hat{\beta}_{\text{GWAS}, j}}{B_{2,j}+(1-p_{\text{causal}})exp\left[ -{C_{2,j}\hat{\beta}}_{\text{GWAS},j}^{2} \right]}$$

And

$$h_{j}^{'}\left( \hat{\beta}_{\text{GWAS}, j} \right)=\frac{A_{2,j}B_{2,j}+A_{2,j}(1-p_{\text{causal}})(1+2{C_{2,j}\hat{\beta}}_{\text{GWAS},j}^{2})exp\left[ -{C_{2,j}\hat{\beta}}_{\text{GWAS},j}^{2} \right]}{\left( B_{2,j}+(1-p_{\text{causal}})exp\left[ -{C_{2,j}\hat{\beta}}_{\text{GWAS},j}^{2} \right] \right)^{2}}$$

We also note that the sampling variance of GWAS effect sizes is approximately

$$\mathrm{var}[\hat{\beta}_{\text{GWAS}, j}]=\frac{1}{2{Nf}_{j}(1-f_{j})}$$

Hence, the sampling variance of the posterior mean of individual SNP effect sizes can be approximated as:

$$\mathrm{var}[\mathbb{E[}\beta_{j}|\hat{\beta}_{\text{GWAS}, j}]]\approx\left[ h_{j}^{'}\left( \hat{\beta}_{\text{GWAS}, j} \right) \right]^{2}\cdot\frac{1}{2{Nf}_{j}(1-f_{j})}$$

Finally, the sampling variance $\mathrm{var}\left[ \hat{d} \right]=\left( s.e.(\hat{d} ) \right)^{2}$ of the posterior mean $\hat{d}$ can be approximated by plugging in this expression for each SNP:

$$\mathrm{var}[\hat{d}]=\sum_{1}^{M} 4\left( f_{j}-g_{j} \right)^{2}\mathrm{var}[\mathbb{E[}\beta_{j}|\hat{\beta}_{\text{GWAS}, j}]]$$

$$\approx\sum_{1}^{M} 4\left( f_{j}-g_{j} \right)^{2}\cdot\frac{\left[ h_{j}^{'}\left( \hat{\beta}_{\text{GWAS}, j} \right) \right]^{2}}{2{Nf}_{j}(1-f_{j})}$$
