# Supplementary material for: Bayesian approach to assessing population differences in genetic risk of disease with application to prostate cancer
Source: PLoS Genet. 2024 Apr 17;20(4):e1011212. doi: 10.1371/journal.pgen.1011212 (PMC11023298; doi:10.1371/journal.pgen.1011212)
Supplement: S4 Appendix — (DOCX) [file pgen.1011212.s004.docx]

## S4 Appendix

## Wright $\boldsymbol{F}_{\boldsymbol{ST}}$ approximation.

Let the allele frequencies for variant $j$ be $f_{j}$ and $g_{j}$ in the population 1 and 2. Define the estimator:

$$\hat{F}=\frac{1}{M}\sum_{1}^{M} \frac{\left( f_{j}-g_{j} \right)^{2}}{2f_{j}(1-f_{j})}$$

Under the Balding-Nichols model [1] for the allele frequencies in each population, we show that the estimator $\hat{F}$ provides an unbiased estimator to Wright’s $F_{ST}$. Assume here that $f_{j}$ are constants with $g_{j}$ modelled as independent random variables. Using a version of the Balding-Nichols model where the ancestral allele frequency is unknown [2] it can be assumed that $g_{j}$ follow the distribution

$$g_{j} \sim\mathrm{Beta}\left( \alpha=\frac{f_{j}\left( 1-2F_{ST} \right)}{{2F}_{ST}},\beta=\frac{(1-f_{j})(1-2F_{ST})}{{2F}_{ST}} \right)$$

Since $\mathbb{E[}g_{j}]=f_{j}$ and$\mathrm{var}\left[ g_{j} \right]={2F}_{ST}f_{j}(1-f_{j})$, note that

$$\mathbb{E}\left[ \left( f_{j}-g_{j} \right)^{2} \right]=\mathbb{E}\left[ \left( g_{j}-\mathbb{E[}g_{j}] \right)^{2} \right]$$

$$=\mathrm{Var}\left[ g_{j} \right]$$

$$={2F}_{ST}f_{j}(1-f_{j})$$

Hence,

$$\mathbb{E}\left[ \hat{F} \right]=\frac{1}{M}\sum_{j=1}^{M} \frac{\mathbb{E}\left[ \left( f_{j}-g_{j} \right)^{2} \right]}{{2f}_{j}(1-f_{j})}$$

$$=\frac{1}{M}\sum_{j=1}^{M} \frac{{2F}_{ST}f_{j}(1-f_{j})}{{2f}_{j}(1-f_{j})}$$

$$=F_{ST}$$

**References**

1. Balding DJ, Nichols RA. A method for quantifying differentiation between populations at multi-allelic loci and its implications for investigating identity and paternity. Genetica. 1995;96(1-2):3-12. Epub 1995/01/01. doi: 10.1007/BF01441146. PubMed PMID: 7607457.

2. Bhatia G, Patterson N, Sankararaman S, Price AL. Estimating and interpreting FST: the impact of rare variants. Genome Res. 2013;23(9):1514-21. Epub 20130716. doi: 10.1101/gr.154831.113. PubMed PMID: 23861382; PubMed Central PMCID: PMCPMC3759727.
