# Supplementary material for: Bayesian approach to assessing population differences in genetic risk of disease with application to prostate cancer
Source: PLoS Genet. 2024 Apr 17;20(4):e1011212. doi: 10.1371/journal.pgen.1011212 (PMC11023298; doi:10.1371/journal.pgen.1011212)
Supplement: S5 Appendix — (DOCX) [file pgen.1011212.s005.docx]

## S5 Appendix

## Simulation study steps for estimating the expected $\mathbf{var}\boldsymbol{[d|}{\hat{\boldsymbol{\beta}}}_{\mathbf{GWAS;}}\boldsymbol{,}\boldsymbol{h}_{\boldsymbol{g}}^{\boldsymbol{2}}\boldsymbol{,}\boldsymbol{p}_{\mathbf{causal}}\boldsymbol{]}$

Our simulation study is implemented based on the following pseudocode steps:

Initialise parameters:

Define $h_{g}^{2}$ and $p_{\text{causal}}$, the number of SNPs, $M$, the population distance $F_{ST}$, and the training sample size, $N$

Set the number of simulation replicates, $B$

**For** $i$ = 1 to number of iterations, $B$

**For** $j$ = 1 to $M$

Simulate $f_{j}$ with uniform distribution on [0.1,0.9]

Simulate $g_{j}$ from beta distribution with parameters $f_{j}(1-{2F}_{ST})/2F_{ST}$ and $(1-f_{j})(1-{2F}_{ST})/2F_{ST}$

Simulate true effect size $\beta_{j}$ from $N\left( 0,h_{g}^{2}/\{Mp_{\text{causal}}\left[ {2f}_{j}\left( 1-f_{j} \right) \right]\} \right)$ with probability $p_{\text{causal}}$, and set to 0 with probability (1$-p_{\text{causal}}$)

Simulate marginal effect estimate $\hat{\beta}_{\text{GWAS}, j}$ from$N\left( \beta_{j}, 1/[2{Nf}_{j}(1-f_{j})] \right)$

**End for**

Calculate $\mathrm{var}[d|{\hat{\boldsymbol{\beta}}}_{\mathbf{GWAS;}},h_{g}^{2}, p_{\mathrm{causal}}]$ using analytical expressions (S1 Appendix), where $h_{g}^{2}$ and $p_{\mathrm{causal}}$ are set to their true initialised values

**End for**

Estimate the expected $\mathrm{var}[d|{\hat{\boldsymbol{\beta}}}_{\mathbf{GWAS;}},h_{g}^{2}, p_{\mathrm{causal}}]$ as the mean across all iterations, $B$
