# Supplementary material for: Bayesian approach to assessing population differences in genetic risk of disease with application to prostate cancer
Source: PLoS Genet. 2024 Apr 17;20(4):e1011212. doi: 10.1371/journal.pgen.1011212 (PMC11023298; doi:10.1371/journal.pgen.1011212)
Supplement: S6 Appendix — (DOCX) [file pgen.1011212.s006.docx]

## S6 Appendix

## Simulation study steps for estimating the bias, sampling variance, and mean square error of $\hat{\boldsymbol{d}}$, and the type 1 and 2 error for the Wald test and $\boldsymbol{t}$-test on $\hat{\boldsymbol{d}}$

Our simulation study is implemented based on the following pseudocode steps. The tolerance $\tau$ in practice was set to 0.01.

Initialise parameters:

Define $h_{g}^{2}$ and $p_{\text{causal}}$, the number of SNPs, $M$, the population distance $F_{ST}$, and the training GWAS sample size, $N_{\text{Training}}$

Define the target sample size for both populations, $N_{\text{Target}\text{,1}}$, $N_{\text{Target}\text{,2}}$ (which in practise we assume are the same size)

Define the genetic log relative risk $d$ = $\text{log}(RR)$

Define the size $\alpha$ of the Wald test

Set the tolerance $\tau$ for the true population log relative risk $d$

Set the number of simulation replicates, $B$

**For** $i$ = 1 to number of iterations, $B$

**For** $j$ = 1 to $M$

**While** $\left| d-d^{'} \right|\geq\tau$

Simulate $f_{j}$ with uniform distribution on [0.1,0.9]

Simulate $g_{j}$ from beta distribution with parameters $f_{j}(1-{2F}_{ST})/2F_{ST}$ and $(1-f_{j})(1-{2F}_{ST})/2F_{ST}$

Simulate true effect sizes $\beta_{j}$ from $N\left( 0,h_{g}^{2}/\{Mp_{\text{causal}}\left[ {2f}_{j}\left( 1-f_{j} \right) \right]\} \right)$ with probability $p_{\text{causal}}$, and set to 0 with probability ($1-p_{\text{causal}}$)

Calculate true population risk difference $d^{'}$ = $\sum_{j=1}^{M} 2(f_{j}-g_{j})\beta_{j}$

**End while**

Simulate marginal effect estimate $\hat{\beta}_{\text{GWAS}, j}$ from$N\left( \beta_{j},1/[2{Nf}_{j}\left( 1-f_{j} \right)] \right)$

**End for**

Calculate posterior mean $\hat{d}\mathbb{=E[}d|{\hat{\boldsymbol{\beta}}}_{\mathbf{GWAS}}\boldsymbol{;}h_{g}^{2},p_{\text{causal}}]$ using analytical expressions (S1 Appendix), where $h_{g}^{2}$ and $p_{\mathrm{causal}}$ are set to their true values

Calculate the sampling variance $\text{var}$[$\hat{d}$] using the delta method (S3 Appendix), where $h_{g}^{2}$ and $p_{\mathrm{causal}}$ are set to their true values

Calculate the Wald statistic ${W=\hat{d}}^{2}/var[\hat{d}]$

Calculate the sampling variance of the $t$-test as $s^{2}\left( \frac{1}{N_{\mathrm{Target},1}}+\frac{1}{N_{\mathrm{Target},2}} \right)$, where $s^{2}=\sum_{j=1}^{M} \left[ 2f_{j}\left( 1-f_{j} \right)+2g_{j}\left( 1-g_{j} \right) \right]\hat{\beta}_{\mathrm{GWAS},j}^{2}$

Calculate the $t$-test statistic as $T=\hat{d}/\sqrt{s^{2}\left( \frac{1}{N_{\mathrm{Target},1}}+\frac{1}{N_{\mathrm{Target},2}} \right)}$

**End for**

Estimate the bias, sampling variance and mean square error of $\hat{d}$ as the sample mean of these performance measures across iterations, $B$

Estimate the power of the Wald test for testing $d$ = 0 as the mean of $1-\Phi(z_{\alpha/2}-\sqrt{W})+\Phi(z_{1-\alpha/2}-\sqrt{W})$ across iterations (where $z_{\alpha}=\Phi^{-1}(1-\alpha)$ is the upper $\alpha$ point of $N(0,1)$)

For the case of $d$ = 0, estimate the type 1 error as the proportion of iterations where $\sqrt{W}>z_{\alpha/2}$. Estimate the type 1 error of the $t$-test as the proportion of iterations where $|T|>z_{\alpha/2}$.
