# Supplementary material for: Bayesian approach to assessing population differences in genetic risk of disease with application to prostate cancer
Source: PLoS Genet. 2024 Apr 17;20(4):e1011212. doi: 10.1371/journal.pgen.1011212 (PMC11023298; doi:10.1371/journal.pgen.1011212)
Supplement: S1 Table — (DOCX) [file pgen.1011212.s007.docx]

**S1 Table**

**Results from case study in UK Biobank of posterior variance formula**

Estimates of the posterior standard deviation s.d.(d) comparing European and African population disease risk for 28 diseases and complex traits from UK Biobank. Posterior s.d.(d) is computed using LDpred2-auto which accounts for the correlation between the pruned SNPs, and also using equation (1) (see main text), which assumes the SNPs are independent.

|  |  | **LDpred2-auto hyperparameters** | | **Posterior standard deviation,** $\text{s.d.}\boldsymbol{(d)}$ | |
| --- | --- | --- | --- | --- | --- |
| **Phenotype** | **Phenotype Code (Neale Lab)** | $\boldsymbol{h}_{\boldsymbol{g}}^{\boldsymbol{2}}$ | $\boldsymbol{p}_{\text{causal}}$ | **LDpred2-auto** | **Equation (1)** |
| Hypertension | 20002_1065 | 0.12 | 0.078 | 0.10 | 0.12 |
| Asthma | 20002_1111 | 0.056 | 0.030 | 0.053 | 0.060 |
| Rheumatoid arthritis | 20002_1464 | 0.0026 | 0.00014 | 0.0018 | 0.0025 |
| Forced vital capacity | 3062_raw | 0.20 | 0.12 | 0.22 | 0.26 |
| College education | 6138_1 | 0.18 | 0.22 | 0.13 | 0.15 |
| Ever smoked | 20160 | 0.076 | 0.13 | 0.099 | 0.11 |
| Snoring | 1210 | 0.063 | 0.12 | 0.092 | 0.10 |
| Sleep duration | 1160 | 0.073 | 0.14 | 0.15 | 0.17 |
| Hair Colour (light brown) | 1747_3 | 0.050 | 0.00080 | 0.028 | 0.030 |
| LDL Cholesterol | 30780_raw | 0.10 | 0.0035 | 0.092 | 0.11 |
| Mean platelet volume | 30100_raw | 0.33 | 0.015 | 0.22 | 0.26 |
| Cholesterol | 30690_raw | 0.11 | 0.0063 | 0.16 | 0.17 |
| HDL Cholesterol | 30760_raw | 0.24 | 0.036 | 0.085 | 0.10 |
| Red blood cell  distribution width | 30070_raw | 0.11 | 0.013 | 0.15 | 0.18 |
| Monocyte count | 30130_raw | 0.10 | 0.021 | 0.039 | 0.045 |
| Bone mineral density | 3148_raw | 0.23 | 0.030 | 0.035 | 0.042 |
| Eosinophil count | 30150 | 0.16 | 0.034 | 0.16 | 0.19 |
| Red blood cell count | 30010_raw | 0.22 | 0.048 | 0.088 | 0.10 |
| High cholesterol | 20002_1473 | 0.041 | 0.013 | 0.041 | 0.048 |
| Triglycerides | 30870_raw | 0.15 | 0.024 | 0.20 | 0.24 |
| High light scatter  reticulocyte count | 30290_raw | 0.071 | 0.019 | 0.054 | 0.063 |
| Glucose | 30740_raw | 0.039 | 0.014 | 0.16 | 0.18 |
| Tanning | 1727 | 0.15 | 0.0024 | 0.094 | 0.10 |
| FEV1/FVC ratio | 20150_raw | 0.22 | 0.12 | 0.17 | 0.21 |
| Allergy eczema  diagnosed | 20002_1452 | 0.0094 | 0.0065 | 0.012 | 0.013 |
| Age of menarche | 2714 | 0.18 | 0.065 | 0.22 | 0.25 |
| Forced vital capacity | 3062_raw | 0.20 | 0.12 | 0.22 | 0.25 |
| Chronotype  (morning person) | 1180 | 0.12 | 0.14 | 0.23 | 0.26 |
