# Supplementary figures and images for: Bayesian approach to assessing population differences in genetic risk of disease with application to prostate cancer
Source: PLoS Genet. 2024 Apr 17;20(4):e1011212. doi: 10.1371/journal.pgen.1011212 (PMC11023298; doi:10.1371/journal.pgen.1011212)

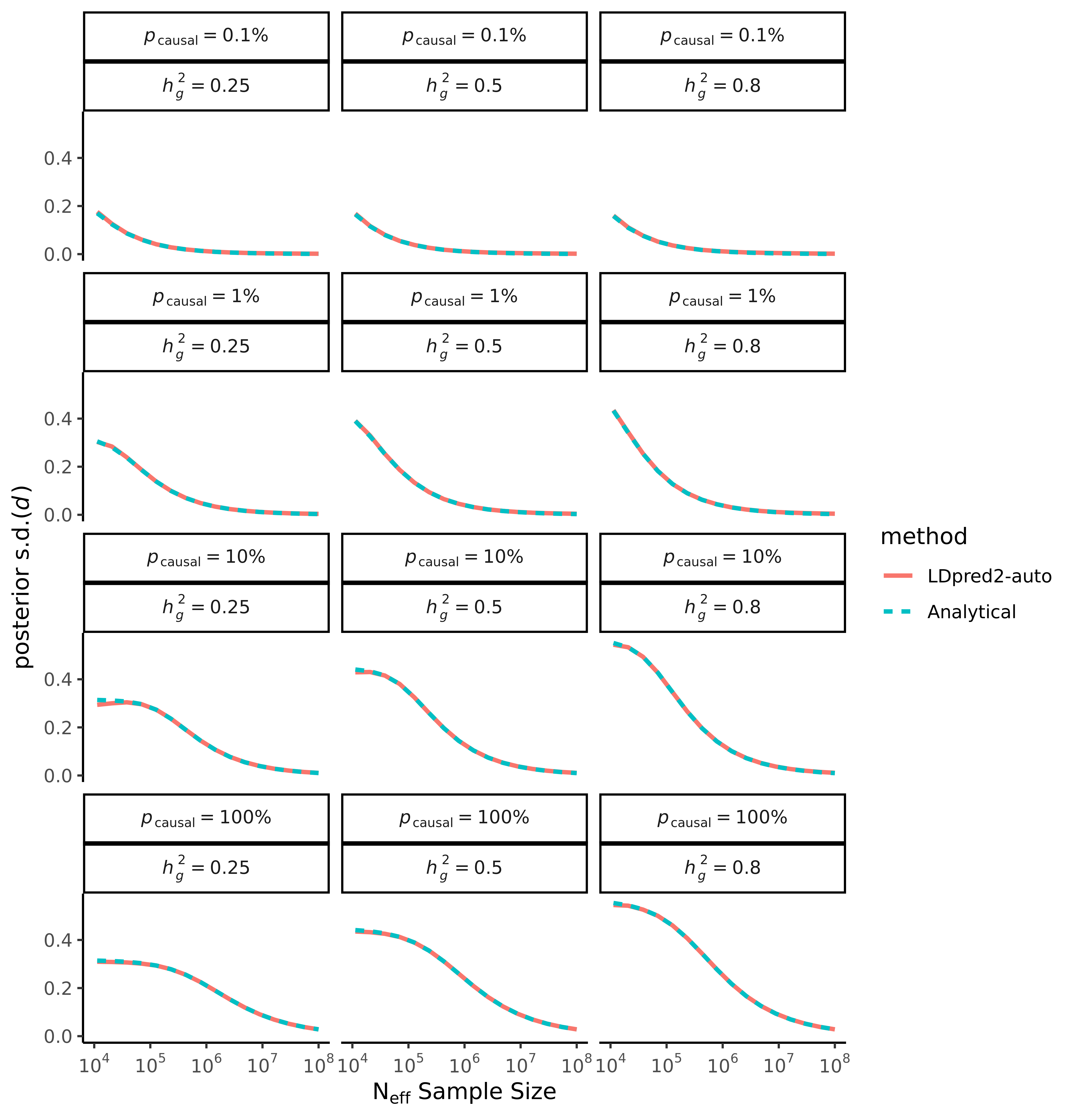

Supplement: S1 Fig — Posterior standard deviation from analytical expressions (Methods and S1 Appendix) compared with estimates from LDpred2-auto using 1,000 MCMC samples after 100 burn-in iterations. FST was fixed at 0.10. Training sample size was varied between 104 and 108. For each set of parameters, the expected posterior s.d.(d) was estimated by averaging across 100 simulations. (TIFF) [file pgen.1011212.s008.tiff]

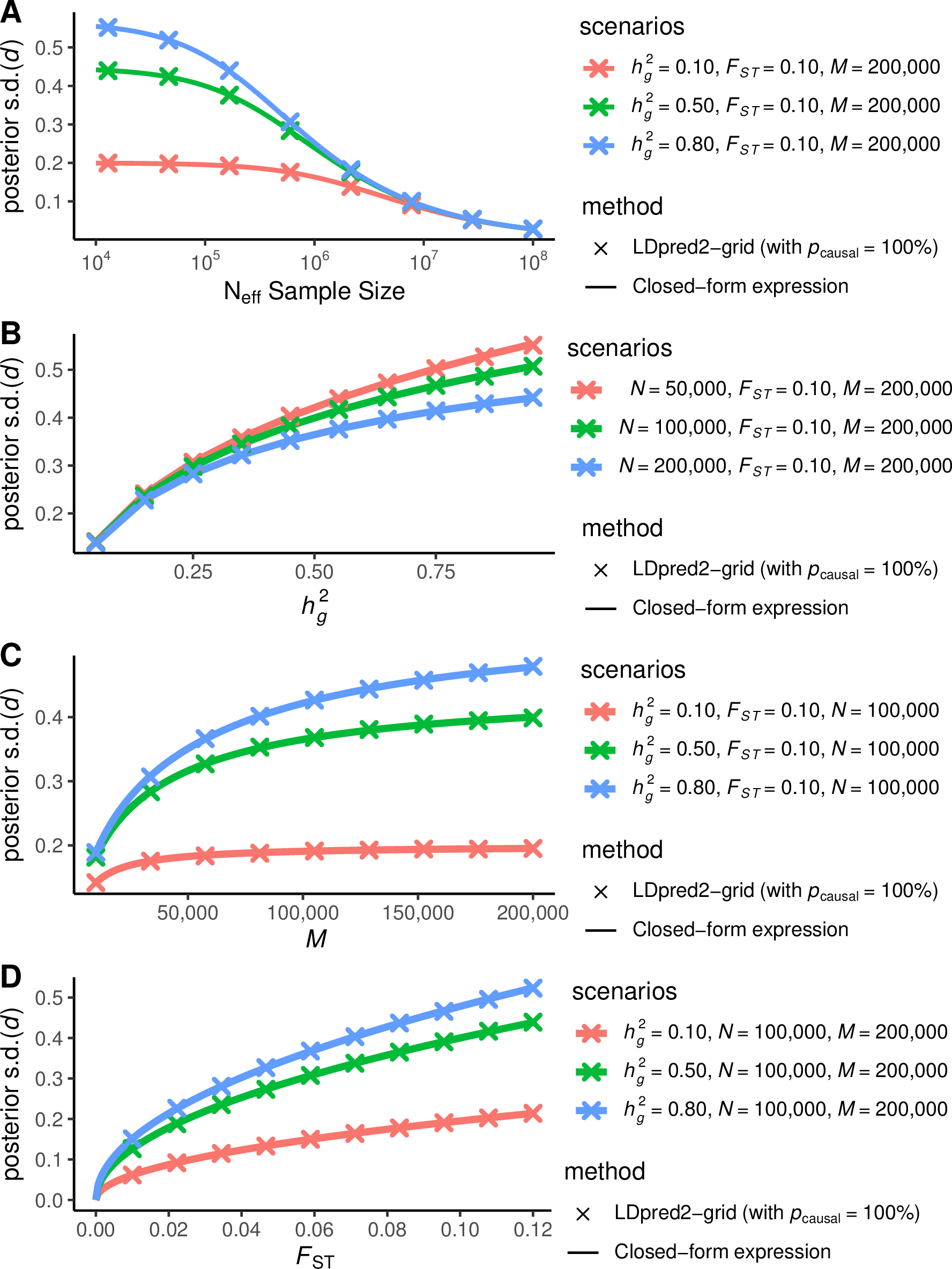

Supplement: S2 Fig — Posterior standard deviation from closed-form expressions for infinitesimal model (S2 Appendix) compared with estimates from LDpred2-grid (with pcausal = 100%) using 1,000 MCMC samples after 100 burn-in iterations. In panels A, B, C and D, each of the parameters N,hg2,M and FST, respectively, are varied while the others are held fixed, with a few scenarios shown in each case. The closed-form expression is plotted as the curved line: var[d]=4MFSTN(1+MNhg2)−1. (TIF) [file pgen.1011212.s009.tif]
