# Supplementary material for: Bayesian approach to assessing population differences in genetic risk of disease with application to prostate cancer
Source: PLoS Genet. 2024 Apr 17;20(4):e1011212. doi: 10.1371/journal.pgen.1011212 (PMC11023298; doi:10.1371/journal.pgen.1011212)
Supplement: S1 Acknowledgements — (DOCX) [file pgen.1011212.s017.docx]

## S1 Acknowledgements

The authors wish to acknowledge the PRACTICAL Consortium:

Rosalind A. Eeles, Christopher A. Haiman, Zsofia Kote-Jarai, Fredrick R. Schumacher, Sara Benlloch, Ali Amin Al Olama, Kenneth Muir, Sonja I. Berndt, David V. Conti, Fredrik Wiklund, Stephen Chanock, Ying Wang, Victoria L. Stevens, Catherine M. Tangen, Jyotsna Batra, Judith A. Clements, APCB BioResource (Australian Prostate Cancer BioResource), Henrik Grönberg, Nora Pashayan, Johanna Schleutker, Demetrius Albanes, Stephanie Weinstein, Alicja Wolk, Catharine M. L. West, Lorelei A. Mucci, Géraldine Cancel-Tassin, Stella Koutros, Karina Dalsgaard Sørensen, Eli Marie Grindedal, David E. Neal, Freddie C. Hamdy, Jenny L. Donovan, Ruth C. Travis, Robert J. Hamilton, Sue Ann Ingles, Barry S. Rosenstein, Yong-Jie Lu, Graham G. Giles, Adam S. Kibel, Ana Vega, Manolis Kogevinas, Kathryn L. Penney, Jong Y. Park, Janet L. Stanford, Cezary Cybulski, Børge G. Nordestgaard, Sune F. Nielsen, Hermann Brenner, Christiane Maier, Jeri Kim, Esther M. John, Manuel R. Teixeira, Susan L. Neuhausen, Kim De Ruyck, Azad Razack, Lisa F. Newcomb, Davor Lessel, Radka Kaneva, Nawaid Usmani, Frank Claessens, Paul A. Townsend, Jose Esteban Castelao, Monique J. Roobol, Florence Menegaux, Kay-Tee Khaw, Lisa Cannon-Albright, Hardev Pandha, Stephen N. Thibodeau, David J. Hunter, Peter Kraft, William J. Blot, Elio Riboli
