# Supplementary material for: Bayesian approach to assessing population differences in genetic risk of disease with application to prostate cancer
Source: PLoS Genet. 2024 Apr 17;20(4):e1011212. doi: 10.1371/journal.pgen.1011212 (PMC11023298; doi:10.1371/journal.pgen.1011212)
Supplement: Attachment — Submitted filename: Responses.pdf [file pgen.1011212.s019.pdf]

Responses to reviewer comments:

Reviewer #1: The authors have addressed most of my concerns. I have some further comments based the author's reply:

1. could you explain the trend of variance in figure 3b, why it goes up and down?

Response: Since the posterior mean is a form of shrinkage estimator, at low sample sizes the shrinkage is artificially high, with estimates made close to zero, which consequently reduces the variance. This comes at the cost of high bias, as we see in the figure (now labelled Figure 4 in the revised manuscript). At high sample sizes, the variance is asymptotically proportional to 1/N, and so decreases toward zero. At intermediate sample sizes there is less shrinkage towards zero, and so the variance is higher. We add clarification on this in the results section at line 275:

> *"… we observed in Figure 4 (B) that the sampling variance of $\hat{d}$ is low at small training sample sizes as the estimates of $\hat{d}$ are drawn towards zero, whilst at the highest training sample size the sampling variance is proportional to 1/N, and between these extremes at intermediate sample sizes the combination of shrinkage and sampling error results in higher sampling variance."*

2. page 18, please clarify that you applied your weights to PRATICAL population, also, if possible, please compute the RR estimated directly from the cohort using the case control data, and compare it with the RR estimated from allele frequency difference.

Response: We have added clarification that weights were applied to the PRACTICAL population on page 18, line 324.

As our data request for the PRACTICAL consortium was restricted to obtaining only summary statistics and allele frequencies, unfortunately we are unable to evaluate our method using individual level case control data. (Access to individual data is

typically restricted to the PRACTICAL study principal investigators, which we are not).

3. could you further comment on how to interpret your results in terms of potential bias of prs as shown in "Testing for differences in polygenic scores in the presence of confounding" https://doi.org/10.1101/2023.03.12.532301

Response: We thank the reviewer for highlighting this potential bias. We agree this is an important effect to consider, and have added the following comment and citation at line 553:

> *'… the impact of other potential sources of population differences, such as non-additive effects [40] and gene-environment interactions [41],* ***and confounding by genetic and environmental effects that correlate with ancestry [42],*** *is unclear.*

4. the structure of the paper looks confusing, for example, it has two results section, please clearly separate results from methods

Response: We are sorry for this confusion. There are results reported in both the 'Verification and Comparison' and 'Applications' sections of the manuscript, which is in line with the structure of Methods papers for this journal. We have tried to improve clarity by avoiding the section title 'Results' in the 'Applications' section, and we have revised the reporting of methods/results around lines 353-359 in the Applications section to improve clarity on this point.

Reviewer #2: - "considering a scenario where all SNPs are independent and all causal SNPs are perfectly recorded, and hence no tagging of causal variants is involved" -> This is not realistic at all; unfortunately, I think this will lead to many false positives. There is more and more evidence that genetic effects are similar across populations.

- "We tried to mitigate this possibility in the prostate cancer data by pruning variants using an African ancestry reference panel" -> On the contrary, I think pruning variants only exacerbates the issue because it makes relying on tagging variants even more.

Response: We accept the point made by the reviewer, and we acknowledge this as an important limitation in the discussion section. We recognise that to address this issue fully we require a thorough investigation of simulations with LD that compare the estimate of $\hat{d}$ from a pruned set of SNPs (or more generally, an imperfectly tagged set of SNPs) with the true d from the full set of causal SNPs. While such simulations can in principle be performed, unfortunately we have been unable to successfully perform them due to limitations with software that we cannot easily resolve. We find there to be substantial convergence issues fitting LDpred2 models to simulated GWAS data with realistic LD patterns. Such issues have been previously reported for simulating realistic data with LD using individual level data (github.com/privefl/bigsnpr/issues/207), though we found such issues also persist when simulating summary statistic data with LD. The added recent features of LDpred2 such as the shrink_corr argument did not help resolve this.

We make a further addition at line 497 (in bold):

> *"Second, our approach depends upon using an independent set of variants to model genetic risk differences, avoiding the complexities of modelling linkage disequilibrium patterns across populations. This however ~~introduces~~* ***can exacerbate the issue of*** *imperfect tagging of causal variants, as information is lost during ~~repeated rounds of~~* ***the*** *pruning ~~across different populations~~* ***process****, …"*

We make a clarification to the discussion at line 505 (in bold):

> *"We **did however try** ~~tried~~ to mitigate **the effect of LD differences** ~~this possibility~~ in the prostate cancer data by pruning variants**, where we used** ~~using~~ an African **(rather than European)** ancestry reference panel for the pruning procedure **to maximise coverage of causal variants**."*

Following on from this, we add a further comment at line 508:

> *"..Nonetheless, we must base our inferences on a set of largely tagged SNPs, and we must further assume that any such allele frequency differences on the tagged SNPs will reflect the differences at the true underlying causal SNPs. Future work should aim to perform simulations and case studies with realistic LD patterns that assess how estimates of population genetic risk differences taken from a set of imperfectly tagged markers, such as the effect estimate on a set of pruned SNPs, compares with the true population difference based on the complete set of causal SNPs."*

Reviewer #3: While the authors have addressed some of my previous comments, several of my previous key concerns were not well addressed. These key concerns are directly related to the practical usefulness of the proposed method. I re-list these key concerns below, with some additional explanations:

1. The main conclusion from the manuscript is that it is important to account for the uncertainty in the SNP effect size estimates when comparing PRS between populations. However, no simulations nor real data results were provided to show that the proposed approach that accounts for such uncertainty works better than the previous approach that directly compares two PRS vectors between populations using a t-test. It is unclear at the moment if one approach provides calibrated type I error control while the other does not, or if one approach is more powerful in detecting PRS differences between population than the other approach. A comprehensive comparison between these approaches should be carried out in both simulations and real datasets to support the conclusion of the paper.

It will be important to directly compare the previous t-test with the proposed Wald test on the inference of the underlying genetic risk. Specifically, you can examine the type I error control of the t-test in settings where there is no difference in the underlying genetic risk, and examine the power of the t-test if there is a difference in the underlying genetic risk. You can then compare type I error and power of the t-test with the proposed Wald test.

Response: We have now provided a head-to-head comparison of the t-test and Wald test within our simulation study, with Figure 4 added to the main text. We have updated S6 Appendix with the steps for performing this simulation. We have added the following summary of the findings to the discussion at line 463:

> *"We note further that the conventional t-test, which compares mean genetic risk differences by evaluating polygenic scores in separate target samples for each population, has very poorly controlled type 1 error rates, even when the initial training sample size is very large. This is due to the t-test detecting mean differences in scores that are due to sampling error in the construction of the polygenic score from the training sample, even when there are no*

underlying true differences in risk across the populations. This effect becomes more severe as the target sample size increases. This is clearly a problematic property of the t-test, and this work provides strong evidence against using this approach. "

4. The main modeling assumption made throughout the manuscript is that the causal effects are the same between populations. With this assumption, the difference in the mean PRS between populations is only due to the differences in causal SNP allele frequencies. However, it is unclear at the moment how realistic this assumption is, given that many previous studies on multi-ancestry modeling make the exact opposite assumption that the causal effect sizes between populations are different. Therefore, it is important to explore how realistic this assumption is in real datasets. It will be important to explore through simulations what consequences are if the causal effect sizes do differ between populations. Ideally, it would be nice if there is a way to quantify the relative contribution of the causal effect size difference and allele frequency difference to the observed PRS difference between populations.
It is key to assess how good this assumption fits the real-world data, as it determines how useful the proposed method is. Assessing this assumption through both simulations and real datasets is needed and is not beyond the current scope of the paper.

Response: It is true that some previous studies have observed moderate causal effect differences across continental populations (Galinsky et al, 10.1002/gepi.22173; Shi et al., 10.1038/s41467-021-21286-1). Yet the most recent studies have now shown that if environments are well controlled, there is minimal heterogeneity in causal effects by ancestry. This includes work by Hou et al. (10.1038/s41588-023-01338-6) and Saitou et al. (10.1101/2022.10.21.22281371) which we have now cited to better justify this modelling assumption. It is reasonable then to attribute observed PRS differences to allele frequency differences. We make the following additions (in bold) and deletions starting from line 516:

"…we have assumed that the causal effect sizes are equal across populations, and hence we focus on differences in disease risk that are

attributable to allele frequency differences (i.e., caused by random drift or selection). ***This is consistent with findings from recent studies which have shown that, when environments are well controlled, there is minimal heterogeneity in causal effects by ancestry [31, 32].*** *~~We consider this a reasonable assumption when the training GWAS data is taken from a large, well-characterised and diverse cross-population meta-analysis, as is the case for prostate cancer and other complex traits and diseases, which are increasingly available.~~ …"*

6. In the simulations, the simulated SNPs are independent of each other in both populations. In the real data, however, SNP independence is achieved through pruning. It would be important to explore realistic simulations with realistic LD patterns, where you can further examine how effective the pruning procedure is in obtaining accurate var(d\hat) estimates.
This is another key issue to address, as it also determines how useful the proposed method is. Assessing this assumption through both simulations and real datasets is needed and is not beyond the current scope of the paper.

Response: After pruning, there remains residual correlation between SNPs, and we can assess whether our variance formulae are sensitive to this, given they assume independence of SNPs. We have now added a further application of our method using summary statistics from 28 phenotypes from UK Biobank. Using a pruned set of SNPs, we compare the var[d] estimates from the analytical formula, which assumes independence, with those from LDpred2-auto, which accounts for correlation. We add the following results to the Application section from line 380:

"We provide a further realistic application of our method to assess the impact of the pruning procedure on the accuracy of s.d.(d). We considered a set of 28 diseases and complex traits from UK Biobank, which we based approximately on sets of phenotypes compiled in previous studies [20, 21], which were representative of a diverse range of genetic architectures…

As expected we observed that using equation (1), which assumes independence between SNPs, there were slightly higher estimates of posterior standard deviation s.d.(d) than those obtained from the LDpred2-auto approach that accounts for correlation between the SNPs. In particular, using equation (1), the estimates of s.d.(d) across 28 phenotypes decreased on average by 13.5% in comparison to LDpred2-auto. This shows that our analytical formula leads to slight overestimates of s.d.(d). Given this finding, our earlier simulation study results (see Verification and Comparison) based on sets of independent rather than pruned SNPs will provide slight overestimates of the true s.d.(d), and are hence mildly conservative regarding the statistical power and type 1 error rates that we would expect in realistic settings."

On the broader related question of how the estimates of d on an imperfectly tagged or pruned set of SNPs compares with the true d on the full set of causal SNPs, we refer to our response to Reviewer 2.
