# Supplementary material for: Bayesian approach to assessing population differences in genetic risk of disease with application to prostate cancer
Source: PLoS Genet. 2024 Apr 17;20(4):e1011212. doi: 10.1371/journal.pgen.1011212 (PMC11023298; doi:10.1371/journal.pgen.1011212)
Supplement: Attachment — Submitted filename: Reviewer responses.docx [file pgen.1011212.s018.docx]

Reviewer's **Comments to the Authors:**

Reviewer #1: Timmins presents an interesting method to compare relative genetic risk across populations while accounting for the uncertainty in PGS estimates. He estimates the genetic risk difference and its uncertainty with the MCMC sampling in LDpred2, which is built on a prior work of Ding et al. The author also developed an analytical form of the uncertainty is genetic risk difference estimate under infinitesimal model. It’s also interesting to see that the Fst between two populations contribute to the significance of genetic risk difference. While I appreciate the concept, major concerns should be addressed prior to publication.

**Response:** We would like to express thanks to the reviewer for their interest in the method, and their time and comments. We have taken the suggestions into account in our revised manuscript.

Major comments:
1. If the author aims to provide a novel tool for testing the significance of genetic risk difference across population, Type I and Type II error should be assessed.

**Response:** We agree that assessing the Type 1 error and statistical power is an important addition. We have now derived a Wald test statistic corresponding to the Bayesian posterior mean estimator $\hat{d}$, and have updated the methods and results sections assessing the properties of this estimator (bias, mse, variance) and corresponding hypothesis test (Type 1 and Power).

We note further that, for this test to be calibrated it needs to be defined using the standard error of $\hat{d}$, rather than the posterior standard deviation of $d$. To do this, we have added further derivations in a new section S3 Appendix which demonstrate how to obtain the standard error of $\hat{d}$. For the non-infinitesimal model this involves use of the delta method (see Efron, doi: 10.1111/rssb.12080).

We have expanded our simulation study to evaluate the properties of $\hat{d}$ and the Wald test, presenting a range of typical scenarios in Figure 3, and the type 1 error rates in supplementary S3 Figure.

Along with revisions to the methods and results, we have added the following paragraph to the discussion:

*“We further derived a Wald test statistic relating to the posterior mean estimator of the log relative risk across populations. We demonstrated that this test statistic had well-calibrated type 1 error rates based on simulations, while the power of the test depended strongly upon the proportion of causal variants along with the training sample size. For more polygenic complex trait genetic architectures (*$p_{\text{causal}}$ *~ 10%), we observed that there was limited statistical power available at current GWAS sample sizes (~100,000) to detect moderate differences in genetic relative risk across populations (relative risk of 1.5 to 2), while to detect smaller differences in risk (relative risk of 1.1 to 1.2) the power only became sufficiently large for sample size more than 1,000,000. In contrast, for less polygenic traits and diseases (*$p_{\text{causal}}$ *~ 1%), there was in general sufficient power available at current available sample sizes to detect more modest differences in genetic risk across populations, as we showed in our prostate cancer example.”*

2. Line 85, is independence assumption required here? I think it’s only useful for deriving the analytical form, since LDpred2 can handle SNPs with LD.

**Response:** The reviewer is correct that the independence assumption is not required here, and so this statement has been deleted. We have made the following additions:

*“Next, we demonstrate that the posterior variance,* $\text{var}[d|\hat{\beta}_{\text{GWAS}},h_{g}^{2}, p_{\text{causal}}]$ *can be evaluated analytically* ***under a simplified assumption where all SNPs are assumed independent****. Under the non-infinitesimal model, we have derived analytical expressions for the posterior variance of individual SNP effect sizes* $\text{var[}\beta_{j}|\hat{\beta}_{\text{GWAS}, j},h_{g}^{2},p_{\text{causal}}]$ *(see S1 Appendix).* ***Using these, and under the assumption of independent SNPs,*** *the posterior variance of* $d$ *can be expressed as…”*

3. For the real data analysis of prostate cancer, more detailed comparison are needed, e.g. provide a table comparing the genetic risk difference estimates from your study and Conti study

**Response:** Table 1 has now been added to compare genetic risk difference estimates across our study and Conti et al. We additionally calculate the statistical power for assessing the prostate cancer risk differences in Table 1.

4. For the example of prostate cancer, it will be interesting to see the relationship between relative risk and Fst. Does the relative risk (or log relative risk) increases linearly with Fat?

**Response:** We haven’t explored the relationship between relative risk and Fst in the prostate cancer example, as it is difficult to ascertain any such relationship across only four populations. Given the form of the posterior variance, we might hypothesis a general relationship across all phenotypes more broadly of the form $\mathbb{E}\left[ d^{2} \right]\propto F_{ST}$, for $d$ = log(RR).

5. There are substantial terminology and mathematical annotations in the main text and supplementary method:
1. Line 81 and 94, what does \tide{beta} mean?

**Response:** We clarify that $\tilde{\beta}$ denotes samples taken from the posterior distribution of $\beta$, which are for example generated using an MCMC/Gibbs sampler. This notation is consistent with Ding et al. (doi: 10.1038/s41588-021-00961-5). We have added the following to the methods:

*“We used* $\tilde{\boldsymbol{\beta}}\sim p(\boldsymbol{\beta}|{\hat{\boldsymbol{\beta}}}_{\text{GWAS}},h_{g}^{2}, p_{\text{causal}})$ *to refer to the samples from the posterior distribution.”*

And:

*“Using the* ***posterior samples*** ${\tilde{\boldsymbol{\beta}}}^{\boldsymbol{(1)}}$*,* ${\tilde{\boldsymbol{\beta}}}^{\boldsymbol{(2)}}$*, …,* ${\tilde{\boldsymbol{\beta}}}^{\boldsymbol{(3,000)}}$***…”***

2. Line 100, 103 and 203 d, instead of \hat{d} because you are describing the variance of d conditional on the data and hyperparameter

**Response:** The reviewer is correct, and this has now been changed. We should further clarify that the notation $\hat{d}=\mathbb{E[}d|{\hat{\boldsymbol{\beta}}}_{\text{GWAS}}\boldsymbol{]}$ denotes the posterior mean, and this is needed elsewhere in the text e.g. for defining the Wald test statistic.

3. Supplementary Line 8, E(\beta_gwas|\beta) is mean, not posterior mean, correct term should be E(\beta|\\beta_gwas)

**Response:** Changed.

4. Supplementary Line 10, same error for posterior variance

**Response:** Changed.

5. Supplementary Line 18, how this \tide beta differ from \hat beta_gwas, if they are the same, please use the same annotation

**Response:** $\hat{\beta}_{\text{GWAS}, j}$ is the correct term here, and this has now been changed. $\tilde{\beta}$ is now only used to denote posterior samples, as described above.

Minor comments:
1. Figure 2, why the analytical form is constant for different polygenicity?

**Response:** This closed-form expression (equation (1), derived in S2 Appendix) is under an infinitesimal model ($p_{\mathrm{causal}}$ = 100%), hence it does not depend on the polygenicity.

In Figure 2 we consider how well the closed-form expression from the infinitesimal model (on the y-axis) approximates the expected true standard error estimated from simulations (on the x-axis) across all genetic architectures (varying $p_{\mathrm{causal}}$).

2. Figure 3, I’m a little confused about the curve, is that the posterior distribution of genetic risk difference? If it’s the case, since the EUR is the base population, shouldn’t the relative risk be deterministic 1?

**Response:** We agree that this is confusing for the reason stated. We had initially aimed to replicate this style of figure from the original Conti et al. paper for comparison. We have now removed this figure and shown the results instead in Table 1.

3. Line 174, I’m confused, is there case/control information in 1000 genome?

**Response:** We apologise for the confusion. There is not to our knowledge any prostate cancer case/control information in 1KGP data. We needed to use 1KGP as a reference panel for pruning to an independent set of SNPs, but the allele frequencies used for comparing population risks were based on the control groups within PRACTICAL study (we didn’t have individual level genotype data from PRACTICAL to perform pruning, hence the 1KGP reference panel was needed).

We have rewritten the methods of this paragraph, with an updated analysis on a wider set of independent SNPs:

*“We estimated the mean genetic risks based on allele frequencies from the control groups of each population. We used a set of 1,444,196 HapMap3+ SNPs [17], which we further filtered to a set of 234,822 approximately independent SNPs through clumping using the African (AFR,* $N$ *= 504) population within the 1000 Genomes project [18] (1KGP), where we clumped based on allele frequency using the bigsnpr R package [19] snp_clumping command with* $r^{2}$ *< 0.1 and 1,000-kb windows.”*

Reviewer #2: The authors use a Bayesian framework to detect differences in genetic effects between populations. While I agree that the authors use a better approach than what is currently performed in practice, I believe that both approaches are not valid. Indeed, shifts in distributions of PGS across ancestries have been shown in multiple papers, and are likely merely due to random drift of allele frequencies (AFs) of tagging variants used in the PGS (i.e. even if the AFs of a causal are the same, the tagging variant used instead may have different AFs across pops). These small differences accumulate and leads to the shifts in distribution, EVEN WHEN THERE IS NO GENETIC DIFFERENCE BETWEEN THE POPS. It would be hard to convince me otherwise. Another alternative would be to rewrite the paper to clearly show the problem I am mentioning, and warn other people about it.

**Response:** The reviewer raises a valid point about the accumulation of imperfect tagging effects, and their potential for biasing estimates of relative risks through shifts in PRS distributions.

To facilitate understanding the uncertainty in relative risk estimates, we have needed to make simplifying assumptions. This has meant considering a scenario where all SNPs are independent and all causal SNPs are perfectly recorded, and hence no tagging of causal variants is involved.

We argue this simplified best-case scenario provides an informative lower bound on the levels of uncertainty expected for these calculations, and this point is currently stated in the discussion. Moreover, using this simplified model it becomes computationally much easier to derive the posterior variance and thus we can simulate large numbers of replicates very efficiently to understand all the appropriate properties of corresponding hypothesis tests (type 1 and 2 error etc.).

Since it is the product of genotype and effect size that contributes to the genetic risk, the risk is the same as if the tagging were perfect but the allele frequencies were different. Thus, imperfect tagging can be captured to a degree by the $F_{ST}$ parameter in our model. We have added a paragraph in the Discussion along these lines.

A more realistic model and accompanying simulations that account for LD and imperfect tagging would certainly build on this work, such as by quantifying the impact on bias in estimates of $d$ and Var[$d$]. We believe however that this would be analytically and computationally very challenging, and we would suggest that this is beyond the scope of our current study.

It is true, however, that in real data any differences in genetic risk identified by our approach could simply reflect differences in LD. We tried to mitigate this possibility in the prostate cancer data through the pruning strategy (Discussion para 7):

“*Differences in genetic risk identified by our approach may simply reflect differences in LD with unknown causal variants. We tried to mitigate this possibility in the prostate cancer data by pruning variants using an African ancestry reference panel.* “

Other comments:
- L122: the equation is missing the LD

**Response:**  We have now added LD to the LDpred2 model description.

- L153: is there any filtering of the chains before averaging?

**Response:** Yes, we have added details on the post-filtering of chains

*“We filtered chains by comparing the scale of the resulting predictions as described in the LDpred2 tutorial vignettes (bigsnnpr, version 1.12.2)”*

- L167-170: you can get the sampling betas directly from LDpred2-auto -> no need for a two-step approach

**Response:** As the reviewer helpfully points out, there is no need for a two-stage approach, as LDpred2-auto betas can be stored using the report_step command. The methods have been updated, and the prostate cancer example has been re-run (with very minor changes to the posterior mean point estimates):

Additionally, the final paragraph of the methods detailing MCMC samples contains duplicated material repeated from earlier in the methods section and has been deleted. We add the following:

*“…* ***Using posterior samples*** ${\tilde{\boldsymbol{\beta}}}^{\boldsymbol{(1)}}$***,*** ${\tilde{\boldsymbol{\beta}}}^{\boldsymbol{(2)}}$***, …,*** ${\tilde{\boldsymbol{\beta}}}^{\boldsymbol{(3,000)}}$ ***(returned using the report_step = 1 argument in LDpred2-auto), we generated posterior samples*** ${\tilde{\boldsymbol{d}}}^{\boldsymbol{(1)}}$***,*** ${\tilde{\boldsymbol{d}}}^{\boldsymbol{(2)}}$***, …,*** ${\tilde{\boldsymbol{d}}}^{\boldsymbol{(3,000)}}$ ***comparing*** *the mean difference in genetic values for European, Hispanic, South Asian and African ancestries...”*

- I am very surprised there are only 36K variants in common

**Response:** We believe that our original pruning strategy within each population was too harsh, and led to surprisingly few SNPs, as noted by the reviewer. We have now updated our prostate cancer analysis to a broader reference panel of hapmap3+ SNPs, which we now only prune in the African ancestry populations using 1KG samples. An updated analysis uses a set of 234,822 approximately independent SNPs. This now aligns better with our simulations, which assume a panel of 200,000 independent SNPs. Our methods section is now updated to the following:

“*We estimated the mean genetic risks based on allele frequencies from the control groups of each population. We used a set of 1,444,196 HapMap3+ SNPs [17], which we further filtered to a set of 234,822 approximately independent SNPs through clumping using the African (AFR,* $N$ *= 504) population within the 1000 Genomes project [18] (1KGP), where we clumped based on allele frequency using the bigsnpr R package [19] snp_clumping command with* $r^{2}$ *< 0.1 and 1,000-kb windows.”*

Reviewer #3: This paper focuses on the problem of identifying the mean difference in the genetic risk scores (PRS) between populations, where the mean difference is denoted as d\hat in the paper. The paper develops a quantitative approach to calculate the variance of d\hat, denoted as var(d\hat). The unique feature of the proposed approach is that it accounts for the uncertainty in the SNP effect size estimates obtained from PRS models. In particular, the proposed approach is based on the assumption that the causal SNP effect sizes are the same between populations. Consequently, the mean difference in PRS between populations can be explained directly by the causal SNP allele frequency differences between populations. As a result, var(d\hat) becomes a function of the causal SNP allele frequency differences and has a closed form analytic solution under the infinitesimal model where all SNPs are causal. The paper carries out some relatively simple simulations to show that the formula appears to work well.

The overall goal of this paper to account for the uncertainty in SNP effect size estimates for PRS applications is important. However, the paper failed to demonstrate the importance or practical relevance of accounting for such uncertainty, as no comparisons were carried out between the proposed approach and the standard approach where such uncertainty is not accounted for. In addition, the simulation results do not support the closed-form analytic formula derived in the paper, even under the completely polygenic architecture based on which the analytic form is derived. The paper is also not clearly written, and I have a hard time understand the detailed simulation steps. My main concerns are listed below:

**Response:** We would like to express thanks to the reviewer for their detailed and constructive comments, which we have taken into account in the revised manuscript.

1. The main conclusion from the manuscript is that it is important to account for the uncertainty in the SNP effect size estimates when comparing PRS between populations. However, no simulations nor real data results were provided to show that the proposed approach that accounts for such uncertainty works better than the previous approach that directly compares two PRS vectors between populations using a t-test. It is unclear at the moment if one approach provides calibrated type I error control while the other does not, or if one approach is more powerful in detecting PRS differences between population than the other approach. A comprehensive comparison between these approaches should be carried out in both simulations and real datasets to support the conclusion of the paper.

**Response:** We thank the reviewer for this helpful comment. For the Wald test that we have now derived, we have assessed type 1 error. (See response above to Major comment (1) from Reviewer 1). We now additionally provide a clearer comparison of the two approaches in the prostate cancer example in our new Table 1. We also assess the statistical power for the prostate cancer example in Table 1.

It is not clear however that a formal comparison can be made between the t-test and Wald test as we have defined it, since they envisage different sample spaces. For instance, it is unclear in the t-test whether allele frequencies are measured in an independent “target” sample or whether they are measured within the training GWAS sample. For example, many studies use 1KGP samples to estimate allele frequencies when performing a t-test/ANOVA calculation. Thus, there are conceptual challenges in defining the sample space and providing a true like-for-like comparison with the t-test. We argue that trying to adapt and generalise our approach further to ensure a fair comparison with the t-test becomes increasingly complex, and risks distracting from our central goal of assessing the impact of the training sample size on uncertainty in relative risk calculations.

We do note that the previous t-test is appropriate for comparing a given polygenic score (e.g., as a risk predictor) across populations, whereas we are concerned about inference on the underlying genetic risk (lines 67 and 449). Viewed this way, the previous t-test and our Wald test are testing different hypotheses, and a direct comparison is inappropriate. We have added to the introduction:

*“In effect, using* $t$*-tests and ANOVA approaches in this manner assumes that polygenic scores measure genetic risk of disease without any measurement error.* ***This would be appropriate for estimating the accuracy of a given polygenic score between populations, such as to assess prospects for risk prediction. However for inference about the underlying genetic risk, we must allow that polygenic scores are derived from a finite sample of GWAS training data,*** *and as shown previously [10-12], genetic risk estimates based on polygenic scores carry large amounts of uncertainty which should be accounted for in subsequent analyses.* ***“***

As we demonstrate that the uncertainty in estimates of genetic relative risk across population is constrained heavily by the training GWAS sample, this provides sufficient proof that the training sample must be considered, and that the t-test cannot meaningfully assess uncertainty by ignoring this.

2. One main issue of the paper is that the closed form analytic solution, shown in the last equation on page 6 (line 103), is not supported by the simulations. The close form solution is derived based on the infinitesimal model with 100% polygenicity. In the main simulations (line 210-215), the close-form solution gives an estimate of 0.4 for var(d\hat). However, the simulations results show that var(d\hat) is 0.48 under 100% polygenicity. Only when the polygenicity reduces to 10%, var(d\hat) reduces to 0.42, which is close to the closed-form solution. The manuscript did not explain or explore what sources may have contributed to such deviation. This is an important gap that should be fully investigated.

**Response:** We thank the reviewer for identifying this discrepancy. The cause of the deviation was due to the specification of the Balding-Nichols model. We have based our derivations (S4 Appendix) on a version of the Balding-Nichols model where the ancestral population allele frequency is unknown. Yet in our simulation study (as described in the methods) we simulated allele frequencies from a model where we assumed the ancestral population was known and followed a uniform distribution. This resulted in different allele frequency distributions that impacted the validity of the simulation approach.

We have now ensured our simulation has the same specification of the Balding Nichols model to our derivations in S4 Appendix. The closed-form solution and simulations now agree on estimates of posterior Var[d].

Figure 1 and Figure 2 have been updated with these new simulation results. (We have additionally corrected Figure 1, which originally plotted points on the variance rather than standard deviation scale, despite the y-axis label being standard devations).

We have made the following change to the methods:

*“…We generated allele frequencies using a version of the Balding-Nichols model [14] where we assume that the ancestral allele frequency is unknown [15]. For each SNP, the allele frequency* $f_{j}$ *in the first population was drawn from the uniform distribution on [0.1,0.9], and the allele frequency* $g_{j}$ *of the second populations was drawn from a beta distribution with parameters* $f_{j}(1-{2F}_{ST})/2F_{ST}$ *and* $(1-f_{j})(1-{2F}_{ST})/2F_{ST}$*.”*

3. Related to the above point, the closed-form solution is a function of sample size (N), causal SNP number (M), F_st, and heritability h_g^2. Do the simulations support the relationship between var(d\hat) and each of these parameters as described in the closed-form solution?

**Response:** We have now added Supplementary S2 Figure, which shows that the posterior variance estimated from MCMC samples taken from LDpred2-grid ($p_{\text{causal}}$ set to 100%) matches closely to the closed-form expression. The closed-form expression has been plotted as a curved line, where we vary one parameter at a time (of $N$, $h_{g}^{2}$, $M$ and $F_{ST}$,) while fixing the other three. This demonstrates using simulations that the closed-form solution is the correct function of these parameters.

4. The main modeling assumption made throughout the manuscript is that the causal effects are the same between populations. With this assumption, the difference in the mean PRS between populations is only due to the differences in causal SNP allele frequencies. However, it is unclear at the moment how realistic this assumption is, given that many previous studies on multi-ancestry modeling make the exact opposite assumption that the causal effect sizes between populations are different. Therefore, it is important to explore how realistic this assumption is in real datasets. It will be important to explore through simulations what consequences are if the causal effect sizes do differ between populations. Ideally, it would be nice if there is a way to quantify the relative contribution of the causal effect size difference and allele frequency difference to the observed PRS difference between populations.

**Response:** The reviewer raises a valid point about the modelling assumptions, and we have acknowledged this in the discussion section: *“we have assumed that the causal effect sizes are equal across populations…We consider this a reasonable assumption when the training GWAS data is taken from a large, well-characterised and diverse cross-population meta-analysis, as is the case for prostate cancer and other complex traits and diseases, which are increasingly available [31]. However, our approach lacks the flexibility to account for population-specific genetic architectures, since differences in causal variants [32-34] and effect sizes [35, 36] may also drive differences in genetic risk. Incorporating parameters such as a cross-population genetic correlation [37, 38] could be one potential approach for future studies...”*

Further assessing this assumption through simulation studies could be a useful future direction for us to consider, though we consider this beyond the current scope.

We also note that differences in effect sizes change the effective $F_{ST}$ between the populations and could thus be indirectly accommodated in our model:

*“Note however that, in our model, it is the product of genotype and effect size that contributes to genetic risk. Thus, if causal effect sizes are truly different, the genetic risk is the same as that from variants with the same effect sizes but different genotype frequencies. The result of different effect sizes, and also of imperfect tagging, is to increase the effective* $F_{ST}$ *in our model, and we can thus accommodate these factors to some extent.”*

5. I do not follow what was done exactly in the simulations. Specifically, when you compute var(d\hat), did you use the 2nd equation on page 6 (line 94) for computation? If so, did you first fit a PRS model (e.g. LDpred2) to obtain the estimated \beta, or did you directly plug in the true beta? If you fitted a PRS model, did you set the hyper-parameters h_g^2 and p_causal to the truth or did you estimate them from the data? Because these two hyper-parameters are estimated in the real data, it would be important to examine through simulations whether the uncertainty in the estimates of these two hyper-parameters influence estimation results on var(d\hat).

**Response:** We have provided further clarifications of the approach used in simulations and have re-written the text in places to improve readability. We have now listed all the simulation study steps in S5 and S6 Appendix.

In the simulations we estimated the betas by fitting the non-infinitesimal LDpred2 model, though rather than using the MCMC simulations with the bigsnpr package, we evaluated the posterior means of the betas analytically using our derived formulas (S1 Appendix). This sped up the simulations significantly, which was important for many of our analyses (e.g., precise type 1 error calculations required ~ 1,000 simulation replicates of each scenario).

The hyper-parameters were set to the truth for the purposes of simplifying the summary statistic simulations. This has been clarified in the methods section with the following addition:

*“We used the derived analytical expressions to efficiently evaluate the posterior mean and variance of* $d$ *(without recourse to MCMC simulations), where we set* $p_{\text{causal}}$ *and* $h_{g}^{2}$ *equal to their true values.”*

We further show that setting the hyper-parameters to their true values is a reasonable assumption and does not impact the estimation results on var[d]. We provide in Supplementary S1 Figure a comparison of the estimates of var[d] from our simulations (where the hyper-parameters are set to the truth) with LDpred2-auto (implemented using bigsnpr, where the hyper-parameters are inferred within the model). There is minimal difference between var[d] across both approaches, showing that estimation of the hyper-parameters does not propagate into var[d].

6. In the simulations, the simulated SNPs are independent of each other in both populations. In the real data, however, SNP independence is achieved through pruning. It would be important to explore realistic simulations with realistic LD patterns, where you can further examine how effective the pruning procedure is in obtaining accurate var(d\hat) estimates.

**Response:** This is valid point raised by the reviewer.

We would firstly say that an assessment of imperfect tagging on variance estimates under realistic LD patterns in the LDpred2 mixture model has been performed in greater detail by Ding et al. (10.1038/s41588-021-00961-5). Nonetheless how this impacts our pruning approach is unclear.

We do however acknowledge that in practise the issue of LD pruning and tagging of causal variants raises further questions of whether we can accurately assess var[d]. We would suggest that designing further simulation studies that assess the accuracy of var[d] would go beyond the current scope as it would raise substantial computational and analytical challenges.

7. There are standard ways to evaluate the accuracy of variance estimates. Specifically, in the simulations, you can obtain d\hat in each replicate and then use multiple replicates (e.g. 100 or more) to compute the variance of d\hat across replicates. On the other hand, you can directly compute var(d\hat) in each replicate based on your derived formula. You can thus directly compare these two different ways of obtaining var(d\hat) to examine if the derived formula is accurate or not. This standard approach of evaluating the accuracy of variance estimates should be used in simulations.

**Response:** We are assured of the accuracy of the variance formulas derived analytically (in S1 Appendix), as we have now compared the results in detail with LDpred2-auto MCMC simulations, as shown in S1 Figure. It is clear the derived analytical results closely match those from MCMC replicates. We have added to the methods section:

*“We demonstrated the validity of our analytical expressions for the posterior variance of* $d$ *through comparisons with LDpred2-auto for a range of genetic architectures and GWAS training sample sizes (S1 Figure)”*
