# Supplementary material for: Bayesian approach to assessing population differences in genetic risk of disease with application to prostate cancer
Source: PLoS Genet. 2024 Apr 17;20(4):e1011212. doi: 10.1371/journal.pgen.1011212 (PMC11023298; doi:10.1371/journal.pgen.1011212)
Supplement: Attachment — Submitted filename: Responses.docx [file pgen.1011212.s020.docx]

**Editor Decision: “**After looking over the revision and your response to previous reviewer comments, we are willing to accept your manuscript provided you add some additional language to the Abstract and Introduction mentioning that your method is only calibrated for dealing with independent SNPs. This will ensure awareness of this issue and perhaps motivate future research in this area.”

**Response:** Additional clarification that the type 1 error control relates to the assumption of independent SNPs has been added to the Abstract (lines 22-24, additions highlighted):

*“We derive a ~~well-calibrated~~ Wald test for formally assessing the difference in genetic risk across populations,* ***which we show to have calibrated type 1 error rates under a simplified assumption that all SNPs are independent, which we achieve in practise using linkage disequilibrium (LD) pruning.****”*

And to the introduction (lines 91-94, additions highlighted):

*“We further derive a Wald test statistic for formally testing differences in genetic risk across populations,* ***which we show to have good control of type 1 errors under a simplified assumption of independent SNPs, which in practise we achieve through a pruning approach. We stress that future research should seek to extend this method further to account for the realistic effects of linkage disequilibrium (LD) and imperfect tagging.”***

There are no further reviewer comments to respond to. We thank the reviewers for their constructive comments throughout, which have much improved the manuscript.
